# Moving on from Metchnikoff: thinking about microbiome therapeutics in cancer

**DOI:** 10.3332/ecancer.2018.867

**Published:** 2018-09-05

**Authors:** Saman Maleki Vareki, Ryan M Chanyi, Kamilah Abdur-Rashid, Liam Brennan, Jeremy P Burton

**Affiliations:** 1 Division of Experimental Oncology, Department of Oncology, Schulich School of Medicine, 1151 Richmond St, London, ON N6A 5C1, Canada; 2 Lawson Health Research Institute, 268 Grosvenor Street, London ON N6A 4V2, Canada; 3 Canadian Centre for Human Microbiome and Probiotics, 268 Grosvenor Street, London ON N6A 4V2, Canada; 4 Division of Urology, Department of Surgery, Schulich School of Medicine, 268 Grosvenor Street, London ON N6A 4V2, Canada; 5 Department of Microbiology & Immunology, University of Western Ontario, 1151 Richmond St, London, ON N6A 5C1, Canada

**Keywords:** probiotic, prebiotic, faecal transplant, oligosaccharides

## Abstract

Precision medicine now needs to also consider the microbiome in oncology treatment. Ingested substances, whether they are a carcinogenic or therapeutic agent, will likely come into contact with the microbiota. Even those delivered extra-intestinally can be influenced beyond xenobiotic metabolism by biochemical factors associated with the microbiota or by an immunological predisposition created by the microbiome. We need to undertake one of the largest paradigm shifts to ever occur in medicine, that is, every drug or ingested substance needs to be re-evaluated for its pharmacological effect post-microbiome interaction. The importance of the microbiome with a focus on the treatment of cancer is discussed. In the near future, it may be possible to specifically manipulate the microbial composition within cancer patients to improve the therapeutic potential of existing oncological agents. However, the current tools to do so are limited. Targeted modulation is likely to be achieved by addition, selective enhancement or depletion of specific microbial types. This may include compounds such as narrow spectrum antimicrobial agents or oligosaccharides that will kill or enhance the bacterial growth of distinct members of the microbiota, respectively. This will stimulate a new era in these fields.

## Introduction

The human microbiome is a complex ecosystem that has coevolved with its host, though our understanding of its wider functional role is limited. We are just starting to appreciate that the metabolic break down of dietary and ingested substances by the microbiota can have a major influence on biological systems. Xenobiotic metabolism is likely to become a major focus of forthcoming microbiome studies as it will change how pharmaceuticals are delivered. This is not an original notion. Reflections of Metchnikoff’s work examining the ‘human flora’, probiotics and his obsession with aging are being revisited. There was a period of time between our understanding of infectious disease and the discovery of the first antibiotics where the balance between microbes and ourselves was strongly considered [1]. Metchnikoff postulated that it was the putrefaction of the colon by toxin-producing bacteria that was involved in its carcinogenesis [2]. We now hail Metchnikoff as the father of microbiome therapeutics but the bacteria themselves are only half the story.

Metchnikoff was aware that he did not have a complete picture but thought that the scientific dots would eventually be joined. With modern techniques, the picture is starting to emerge. While his chapter entitled ‘disharmonies of digestion’ is a spoiler for his clear disdain for the colon and non-milk-souring bacteria, it was his observations which led him to describe ‘the useless of the intestine’ and reach his conclusions. For instance, he observed a woman who had lived for 37 years without a functioning colon due to a caecal–rectal fistula. Metchnikoff believed that the large intestine was likely inherited through our vegetarian ancestors to function as a storage reservoir for waste products, similar to that of the urinary bladder. With a poor diet, he considered it as an organ waiting for disaster and predisposed humans to diseases like cancer. It was the belief that perhaps led him to become the first ‘germophobe’. Lankester, a friend who wrote the preface of his biography, speaks of Metchnikoff’s refusal to eat any uncooked salads, unpeeled fruits or anything that may contain bacteria [3]. Metchnikoff theorised that there was a link between the consumption of ‘soured milk’ and the life expectancy of Bulgarian peasants. This was controversial, but current science is diving in, separating myth from microbes and health. The reality is that longer lives allow cancer to become a more prevalent cause of mortality in developed countries. This is in contrast to the past where life expectancy was decreased largely due to infectious disease. Humans are trying to fine-tune their understanding of where they exist in the ‘biological environment’, which includes the microbes within it.

People that consume microorganisms, in the form of fermented foods may have long-term health benefits, leading healthier lives and experiencing less disease [4]. If microorganisms have a role, it is prudent to understand which ones and if there are specific species or strains responsible. Even the simplest of animal models, such as *Drosophila melanogaster*, require the consumption of bacteria from the environment, without which they perform more poorly and have shorter lives [5]. While animal models have microbiomes, they are quite different from humans, and therefore observations may not fully translate between humans and other species [6]; there is still much that can be learned in the context of the microbiome’s importance to humans. It is difficult to reliably track microorganisms within complex microbiomes. Next-generation sequencing of the 16S rRNA gene has greatly increased the understanding of the microbiome and the broader compositional characteristics of the ecosystem. However, it can only describe which microorganisms are present but not what function/role, if any, they play. The reality is that using these techniques, most bacterial types can only be identified to the genus level, at best. Considering that there are substantial genetic and functional differences among different strains of the same species, any functional assumptions on what these bacteria are doing is limited. Scientists are at the beginning of the next era of metagenomic and metabolomic studies that will be able to answer many of these important questions.

Many studies of the intestinal microbiome observe a decline in bacterial diversity with age or disease [7]. This has also been confirmed in animal models [8]. Our laboratory has recently completed a collaborative study in China [9], where the gut microbiota was examined of a cross-sectional cohort of more than 1,000 very ‘healthy’ Chinese people ranging from 3 years of age to centenarians over the age of 100. One of the most interesting results was the finding that the microbiota composition of the centenarians was similar to that of people decades younger, 30–50 years old, in contrast to other studies. This raises the usual questions. Is the microbiome only reflective of its host’s physical state? Or has it played a protective role in keeping the individual disease free? The renewed fascination with the microbiome will certainly drive studies looking at its diagnostic (prognostic and predictive) and treatment potentials for some time.

## Understanding metabolism of drugs

There are two important factors to understand regarding the metabolism of drugs by bacteria. First, many pharmaceutical agents have poor solubility, and second, microbes can utilise a diverse source of nutrients. Due to ease of administration, oral ingestion is the most common and preferentially employed delivery route for pharmaceuticals because it is associated with high patient compliance, cost-effectiveness and low sterility concerns, among others [10]. Modest bioavailability is the major obstacle for the oral dosing regimens of drugs. This is largely attributed to poor solubility and low gut permeability [10]. Therefore, drugs with poor solubility often require high doses to reach therapeutic concentrations in serum. It is estimated that more than 40% of new chemical entities developed in the pharmaceutical industry are practically insoluble in water [10].

Microbes exist in the most inhospitable environments on this planet and use the most diverse substrates as carbon and nitrogen sources to survive. The microbes that exist within us have an immense capacity of metabolic potential. This is assessed through the metabolic pathways encoded within the genomes of the bacteria found in the gut, which is touted to be over 100-fold the functional capacity of our own capabilities [11]. It is of no surprise that when bacteria encounter a new substrate not typically found within the gastrointestinal tract, many can readily adapt to utilise it. The ability for a bacterium to adapt provides a competitive advantage in a niche abundant with competitors and limited for substrates. While there is an overlapping set of microbes between individuals, each harbours a unique microbiome in some way. It is this diversity that will require management with regard to personalised medicine approaches to ensure the appropriate response with a pharmaceutical agent. It will be required to know which microorganisms are present not only as a measure of likely xenobiotic activity but also for gastrointestinal mucosal toxicity.

## Microbes and medicines

There is a shift in focus towards ‘microbiome medicine’, in which the interaction between the microbiome and medicine needs to be taken into consideration [12]. The cardiac drug digoxin was the first pharmacological agent that was identified to be influenced by bacteria within the gut. The bacterium *Eubacterium lentum* was a common inhabitant of the microbiota and could reduce the lactone ring of digoxin. This inactivated the compound and produced nephrotoxic metabolites [13]. This finding launched an entirely novel field of microbial and pharmaceutical research. It was unknown what other bacteria could metabolise pharmaceutical agents and what effect this has on the drug or the patient. The efficacy of cancer alkylating agents (cyclophosphamide) and platinum salts (cisplatin) can also be affected by the microbiome. In germ-free mice, where no bacteria are present within the mouse, the efficiency of these compounds was greatly reduced [14]. This same decrease in efficacy was also recapitulated in other animal models given antibiotics to remove bacteria in the gut. Doxorubicin is widely used in cancer treatment and was originally isolated from specific strains of the bacterium, *Streptomyces peucetius var. casieus*. Another strain of *Streptomyces*, simply called WAC04685, was found to inactivate it via a reductive deglycosylation mechanism [15]. Later, *Raoultella planticola*, *Klebsiella pneumoniae* and *Escherichia coli* were all found to inactivate doxorubicin via a similar pathway [16]. Although doxorubicin is a compound delivered by intravenous injection, the finding demonstrates the ability of many microorganisms to break down pharmaceutical compounds. While these examples are of bacteria breaking down drugs, removing their activity, it hints at the idea of microbiome therapeutics. It has already begun where there are biologically inactivated compounds that require the microbiome to modify them to become active once ingested. In the future, these will play a role in mainstream anticancer therapeutics. A recent study proposed the TIMER framework, which outlines several mechanisms where chemotherapeutic agents having adverse effects on the gut microbiota modulates downstream responses, often leading to poorer outcomes [17].

There is a lot of redundancy in the metabolic pathways that are encoded among different microbes. Within these pathways are some that encode functions that are rarely required by the organisms in the host. For example, specific nutrient exploitation (e.g. oxalate), antibiotic resistance, heavy metal utilisation/resistance and the production of hormones, such as androgens, by very selected microbial populations [18, 19]. When compounds not typically present in our diet are ingested, those capable of utilising it are quickly enriched. This rapid shift in the microbial composition can be observed in the laboratory using chemostat models of the digestive tract [20]. *In vivo*, this change could have important consequences for the host as medications intended for a disease may be modified by the microbiome such that it is inactivated or may become toxic to the host. Some commonly prescribed medications are already known to do this; however, that the vast majority of all pharmaceutical compounds have not been investigated. For example, metformin is a commonly prescribed drug for diabetes and has been recently found to modify the microbiome [21]. This may explain certain side effects, such as diarrhoea, through the promotion of *E. coli*. It may also explain certain benefits as well, as the intravenous administration does not improve glycemia [22]. *Akkermansia muciniphila* is a bacterium known to reverse metabolic disorders and is also positively associated with metformin use [23]. Metformin is also being repurposed beyond diabetes as a cancer therapeutic, with the thought of its ability to arrest metabolic activities [24, 25]. Interestingly, several meta-analysis studies have shown an association with the decreased risk of the occurrence of various types of cancers including colorectal cancer in type 2 diabetic patients taking metformin. However, the exact mechanism of such an effect is not understood [24]. Given that the microbiome is linked to the control of insulin and this has some influence on prostate cancer growth, a microbial role in this mechanism would be worthy of investigation.

One of our group’s current interests is the drug Zytiga (abiraterone acetate) that is used to treat metastatic castration-resistant prostate cancer. Androgens and their receptors play a key role in the progression of prostate cancer. Simply, testosterone-depleting drugs target biosynthesis (e.g. abiraterone acetate) or outcompete testosterone at androgen receptors (e.g. enzalutamide). These compounds are taken orally and some have poor solubility, hence spending a considerable amount of time in the presence of bacteria in the gut. This maximizes the likelihood for their xenobiotic modification. The acetate portion of abiraterone acetate is a possible carbon source that could be used by microorganisms. There are at least two pathways of importance when examining the xenobiotic metabolism of any drug. First, the microbiome may modify the drug into a more active form or inactivate the drug altogether. Second, the microbiome may produce metabolites to mitigate the effect of drugs, such as the regulation of testosterone in prostate cancer. This may act as a redundancy mechanism in the absence of endogenous production of testosterone; however, it may prove detrimental in the context of prostate cancer patients. This situation is unusual with regard to physiology for men with prostate cancer, as changes due to the rapid and almost complete depletion of the major sex hormone may place additional selective pressure on bacteria to compensate for the unavailability of certain molecules and change in conditions. Recent studies in murine models show a role in testosterone regulation by gut bacteria [26, 27]. Further mechanisms of exploitation of the sex hormone regulation by the microbiome are likely since these organisms play a role in other cancers such as breast cancer.

While microbiome studies are primarily gut centric, chemotherapeutic agents may affect bacteria at other sites as well. Milk samples collected from a lactating woman undergoing chemotherapy for Hodgkin’s lymphoma changed dramatically in microbial composition, deviating from what is thought to be a healthy microbial profile [28], a worrying factor for microorganisms found directly in breast cancer [29, 30]. The support to patients either in the form of pro- or pre-biotic supplementation is likely to have considerable benefit in reducing side effects by mitigating microbial factors.

## Improving response to immune checkpoint inhibitors by modifying patient’s microbiome

It is well established that the human microbiome can play a major role in shaping the immune system. Therefore, it is perhaps not too surprising that the microbiome can also impact a patient’s response to immune checkpoint inhibitors (ICIs) that primarily target the immune system rather than the patient’s tumour. Drugs that target programmed death 1 (PD-1) or its binding partner PD-L1 are becoming mainstream in oncology. Recently, a seminal study by Routy *et al* [31] revealed that gut microbiome could affect the efficacy of ICIs in solid tumours [31]. Interestingly, colonising germ-free mice or antibiotic-treated animals with the faecal microbial transplant (FMT) from non-small cell lung cancer patients that responded to ICIs, as oppose to nonresponders, sensitised animals to anti-PD-1 therapy [31]. This effect was accompanied by increasing the number of tumour-infiltrating lymphocytes (TILs) [31]. Importantly, these results were reproducible in other models [31]. These are critical observations because: a) it demonstrates that transferring microbiota via FMT from a donor responding to ICIs can render another host responsive to a similar treatment and b) such an effect is likely because of increased frequency of TILs into a tumour. This could help to overcome an important hurdle in treating patients with non-T-cell-inflamed tumours, who often show primary resistance to ICIs because of the lack of TILs in their tumours [32].

A separate study by Gopalakrishnan *et al* [33] uncovered that metastatic melanoma patients who responded to anti-PD-1 therapy had a diverse gut microbiome compared to patients that did not benefit from the therapy. Diversity is a well-recognised factor for a healthy microbiome [9]. This can be lost in melanoma patients who do not respond to immunotherapy [33]. Therefore, establishing a diverse gut microbiome in melanoma patients via FMT from a healthy donor with a diverse microbiome profile could improve patient’s response to immune-modulating treatments such as anti-PD-1 therapy.

Note that the antibiotic treatment for common dental, pulmonary and urinary infections significantly reduces progression-free survival and overall survival in patients treated with ICIs [31]. Administering antibiotics can impair therapeutic outcomes of cancers treated with chemotherapy drugs such as cyclophosphamide by dampening endogenous T cell responses and impair adoptively transferred CD4+ T-cells used in immunotherapy [34]. These major observations underline the significance of the microbiome in patients’ response to these immune modulating agents. Perhaps, the negative effect of antibiotics on useful gut microbiota and reducing microbial population diversity are the factors associated with such an important observation. Therefore, clinical studies are required to assess whether using microbiome-replacing methods such as FMT or the use of probiotics could improve patient outcome in response to immunotherapy when a cancer patient requires antibiotic treatment. It should be noted that probiotics often do not colonise and replace the gut microbiome [35] but could be considered as a transient solution when a patient on immunotherapy is required to undergo antibiotic treatments. These observations support the notion that altering the gut microbiome in cancer patients can improve their response to ICIs. Clinical studies are warranted to examine whether the combination of FMT and ICIs or probiotic treatment and ICIs would enhance the efficacy of these immunotherapy agents in cancer patients without added toxicity.

## Microbiome therapeutics beyond soured milk

If it can be assumed that the microbiome plays a part in patient health during a cancer journey from prevention to treatment, then which of the thousands of strains of bacteria are required for the best outcome needs to be determined. Perhaps, a personalised consortium of bacteria unique to the individual and their disease condition would be required for the best patient outcome. Moreover, the microbiome of healthy people is not similar to that of patients, which leads to the question: How will patients potentially respond to medications if there is a microbiome component to the response? Importantly, those with other comorbidities typically receive a multitude of medicines, all of which may shape the microbiome in some way and its net physiological response.

To change the microbiome, microorganisms can be added (probiotics and FMT), the behaviour of existing microbes can be modified (e.g. growth by oligosaccharides and microRNA), the composition can be altered (prebiotics), or those not wanted can be depleted (antimicrobials). The repertoire to do this at present is both crude and limited [36]. However, there will come a time when these approaches can be more targeted, for example, it will be possible to increase the growth of a selected group of organisms by administering a highly specific oligosaccharide prebiotic that only specific microbes can use or with very narrow spectrum antimicrobials.

It is not trivial to change the microbiome in a controlled way due to the synergistic complexity among organisms supporting the growth of each other, host factors and bacterial antagonisms. First, targeted approaches to instil a ‘missing microbe’ are quite hard. Ignoring current regulatory and safety concerns of ‘novel’ species, even most commercially used probiotic strains delivered orally do not usually persist, colonise or even change the microbial composition. There is currently a deficit of knowledge in delivering a microorganism in a product that has the exact same properties of freshly isolated or the bacterium *in vivo*. The characteristics of the same strain (stability, adherence and physiological properties) can be dramatically altered by propagation, fermentation, stabilisation and dosing [37]. While the efficacy of some of these therapies has been shown in well-designed clinical studies, it is assumed on their journey through the intestinal tract, it is the small molecule portion of the microbial fermentate that elicits the biological effect on the host via cell signalling or immunological response. There is no doubt that probiotics have efficacy in certain situations and appear to be good at improving situations such as gut permeability issues that are associated with various chronic diseases as demonstrated in animal models [38] and may be of use in human models of inflammatory bowel disease.

FMTs are widely reviewed elsewhere, but these do offer the opportunity to transfer a more substantial part of an ecosystem synergistically reliant on each other, even though studies suggest that it may just be a component of it and can result in a long-term change in the host’s microbial composition [6]. Such transplants may be useful to obtain microorganisms with potential metabolic pathways or as discussed earlier to improve a patient’s response to immunotherapy against cancer.

## Forgotten antibiotics

With more microbiome information, it may be possible to selectively target groups of bacterial types that may be implicated in the progression of cancer or those that affect the treatment. Antibiotic development has largely focussed upon agents with more broad-spectrum abilities. This has been the case for multiple reasons including to accommodate the clinical need for empiric treatment of conditions before the reliable diagnostic information is obtained from the laboratory. The net result is that antibiotics cause a substantial amount of collateral damage to other members of the microbiota not involved in the disease process with potentially serious adverse health consequences for the host. Antibiotics are generally produced by other microorganisms and there are in fact numerous antimicrobial molecules, especially bacteriocins [protein-based antibiotics] that have potential but were not commercialised due to their narrow spectrum of activities [39]. It seems that if parts of the microbiome can be selectively targeted, there may be a large demand for narrow-spectrum agents to prevent the bystander damage associated with the use of more general purpose antimicrobials. It is unclear whether this would yet make commercial sense, as for every antibiotic that has reached clinical use, exponentially more were culled at development stages and it certainly would not be feasible yet.

There are also other tools that are evolving which may allow a more fine-tuned modification of the microbiome. There is a line of thought that humans select and control their own microbiome through specific receptors and proteins such as human-derived antimicrobial proteins. New findings also show there may be the potential to modify bacterial gene expression by human microRNAs shown to change the bacterial behaviour [40, 41]. There is also recent work that demonstrates that metabolic niches in the microbiota can be exploited to facilitate strain engraftment [42]. These have a great potential for the use as microbiome therapeutic agents as they may provide the potential for highly targeted interventions.

## Useful functional foods

Epidemiological studies looking at a large number of people have shown the immense importance of diet in cancer [4]. Scientists are starting to realise that every ingestible substance, whether drug or food, may have a role in health. Foods with biological functions beyond nutrition are vast and not the focus of this review. Worthy of quick mention, however, due to their biological evolution to specifically modulate bacteria, are the oligosaccharide prebiotics. There are hundreds of these in nature that may promote or inhibit bacterial types in both broad and specific ways. In our need to selectively modify microbiomes with drug treatment, this class of sugars offers a potential mechanism. While these are naturally occurring sugars in foods (chicory, onions and milk), an individual’s diet obviously plays a major role with regard to their intake, although most adults consume them in an *ad hoc* way with or without any true understanding of their net biological contributions. These nutrients are important in modulating bacteria for obesity and other risk factors associated with cancer [43].

Breast milk is potentially a treasure trove of useful molecules as it contains hundreds of oligosaccharides, as well as antimicrobial peptides and antibodies, which selectively promote certain microbial types in the infant (e.g. selected bifidobacterial). These have evolved specifically for this purpose, as many are indigestible to humans [44]. Although it is not feasible for adults to consume components of human milk, there are pure derivatives of these products that are synthesised via engineered means and are reaching the consumer market. These products resemble the natural form giving scientists the opportunity to test their microbiome modifying potential, as well as other therapeutic effects [45]. Given the spectrum of oligosaccharides that exist in human and other sources of milk alone, not to mention those of plant origin, there is great potential to manipulate the microbiome once a better understanding of each oligosaccharide’s specific functional potential is revealed.

## Conclusion

This niche will be a major area of research intensity in the future, but the field still suffers from ‘sour milk syndrome’ whereby significant numbers of both clinicians and scientists discount the role of the microbiome in health and disease. However, while the genome is almost identical among humans, what separates us the most is the composition of the microbiome within us. Each is unique to ourselves as individuals. With this in mind, the gut microbiome has the potential to drastically affect drug responses among individuals, and a better understanding of how to manipulate this ‘organ’ in order to achieve the desired therapeutic response is needed. It is likely that in the future, the microbiomes of patients can be characterised in order to determine if a drug will be effective or not prior to commencing treatment. Studying and understanding the gut microbiome could lead to the development of better therapeutics, alluded to many years ago by great thinkers like Metchnikoff.

## Conflicts of interest

The authors have no conflicts of interest to report.

## Funding

The authors did not receive any funding for this work.
